# Comparative methylomics between domesticated and wild silkworms implies possible epigenetic influences on silkworm domestication

**DOI:** 10.1186/1471-2164-14-646

**Published:** 2013-09-23

**Authors:** Hui Xiang, Xin Li, Fangyin Dai, Xun Xu, Anjiang Tan, Lei Chen, Guojie Zhang, Yun Ding, Qiye Li, Jinmin Lian, Andrew Willden, Qiuhong Guo, Qingyou Xia, Jun Wang, Wen Wang

**Affiliations:** 1 State Key Laboratory of Genetic Resources and Evolution, Kunming Institute of Zoology, Chinese Academy of Sciences, 32 East Jiaochang Road, Kunming, Yunnan Province 650223, China; 2 State Key Laboratory of Silkworm Genome Biology, Key Sericultural Laboratory of Agricultural Ministry, Institute of Sericulture and Systems Biology, Southwest University, No.2 Tiansheng Road, Chongqing, BeiBei District 400715, China; 3 BGI-Shenzhen, Shenzhen, China, Beishan Industrial Zone, Shenzhen, Yantian District 518083, China; 4 Shanghai Institute of Plant Physiology and Ecology, Chinese Academy of Sciences, 300 Fenglin Road, Shanghai 200032, China

**Keywords:** *dnmt1*, Comparative methylomics, Silkworm, Domestication

## Abstract

**Background:**

In contrast to wild species, which have typically evolved phenotypes over long periods of natural selection, domesticates rapidly gained human-preferred agronomic traits in a relatively short-time frame via artificial selection. Under domesticated conditions, many traits can be observed that cannot only be due to environmental alteration. In the case of silkworms, aside from genetic divergence, whether epigenetic divergence played a role in domestication is an unanswered question. The silkworm is still an enigma in that it has two DNA methyltransferases (DNMT1 and DNMT2) but their functionality is unknown. Even in particular the functionality of the widely distributed DNMT1 remains unknown in insects in general.

**Results:**

By embryonic RNA interference, we reveal that knockdown of silkworm *Dnmt1* caused decreased hatchability, providing the first direct experimental evidence of functional significance of insect *Dnmt1*. In the light of this fact and those that DNA methylation is correlated with gene expression in silkworms and some agronomic traits in domesticated organisms are not stable, we comprehensively compare silk gland methylomes of 3 domesticated (*Bombyx mori*) and 4 wild (*Bombyx mandarina*) silkworms to identify differentially methylated genes between the two. We observed 2-fold more differentiated methylated cytosinces (mCs) in domesticated silkworms as compared to their wild counterparts, suggesting a trend of increasing DNA methylation during domestication. Further study of more domesticated and wild silkworms narrowed down the domesticates’ epimutations, and we were able to identify a number of differential genes. One such gene showing demethyaltion in domesticates correspondently displays lower gene expression, and more interestingly, has experienced selective sweep. A methylation-increased gene seems to result in higher expression in domesticates and the function of its *Drosophila* homolog was previously found to be essential for cell volume regulation, indicating a possible correlation with the enlargement of silk glands in domesticated silkworms.

**Conclusions:**

Our results imply epigenetic influences at work during domestication, which gives insight into long time historical controversies regarding acquired inheritance.

## Background

In contrast to wild species that typically evolved phenotypes over a long period of natural selection, domesticates rapidly gained human-preferred agronomic traits under a relatively short-time frame via artificial selection. Under domesticated conditions, it is often to see many obtained traits instable to environmental alteration. The mechanisms underlying these distinct phenomena are not a new issue—as far back as the 1860s, with no knowledge of Mendelian genetics, Darwin speculated such mechanisms underlying the environment-induced changes [1], and in the extreme, this conjecture fits Lamarck’s idea of acquired inheritance. While under many circumstances epigenetically influenced agronomic traits are not so stable as to genetically controlled ones [2,3], many case studies have shown that epigenetic alterations could promptly respond to environmental signals independent of genetic bases [4,5] and differentially methylated alleles could lead to heritable phenotypic changes across generations [2,6-8], including some agronomically important traits [2]. Therefore, as suggested by Hauden et al. [9], we suspect that epigenetic changes might partially account for phenotypic adaptation to the rapid artificial selection.

Silkworms have a relatively short history of domestication, (about 5000 year) but show dramatic phenotypic changes. Genetic divergence between the two species is, of course, an important mechanism of this evolution, which has been assessed by Xia et al. [10]. For the ~14 and ~13 million single-nucleotide polymorphisms they identified in domesticated and wild silkworms, nearly 20% and 15% are species specific and majority of them exist in both species. Similar pattern was also found for indels. Totally 2.9% of the domesticated silkworm genome was detected with selection signals [10]. These results indicate that, although the domesticated silkworms have maintained many genetic variations existing in wild silkworms, they have also clearly genetically differentiated from wild ones. Besides the genetic divergence, whether epigenetic divergence play roles in silkworm domestication, is a new emerging issue in the era of epigenetics. Due in large part to its comparatively small genome (480 Mb) [11], silkworms provide us a special opportunity to study possible DNA methylation influence on domesticated animals in a manner that would be difficult in more genome-complicated animals that have been domesticated. Although we recently demonstrated that methylation in silkworm genes is positively correlated with gene expression levels [12], it is still an enigma if the only two DNA methyltransferase (DNMT1 and DNMT2) existing in silkworms are functional. In insect it has been thought that DNA methylation doesn’t function because in the long-term insect model organism *Drosophila* there is only DNMT2 and DNA methylation seems not functional [13]. Recently Kucharski et al. [14] demonstrated that honeybees have complete DNA methyltransferase set (DNMT1-3) and knock-down *Dnmt3*, one important methyltransferase in animals and plants [15], can make majority of newly hatched larvae emerged as queens, suggesting functional importance of honeybee DNMT3. However, functionality of the more widely distributed DNMT1 (Additional file 1: Figure S1), which is essential for developmental normality and critically required for transgenerational stability of mammals’ and plants’ epigenomes [16,17], remains unknown in insects.

## Results and discussion

**Figure 1:**
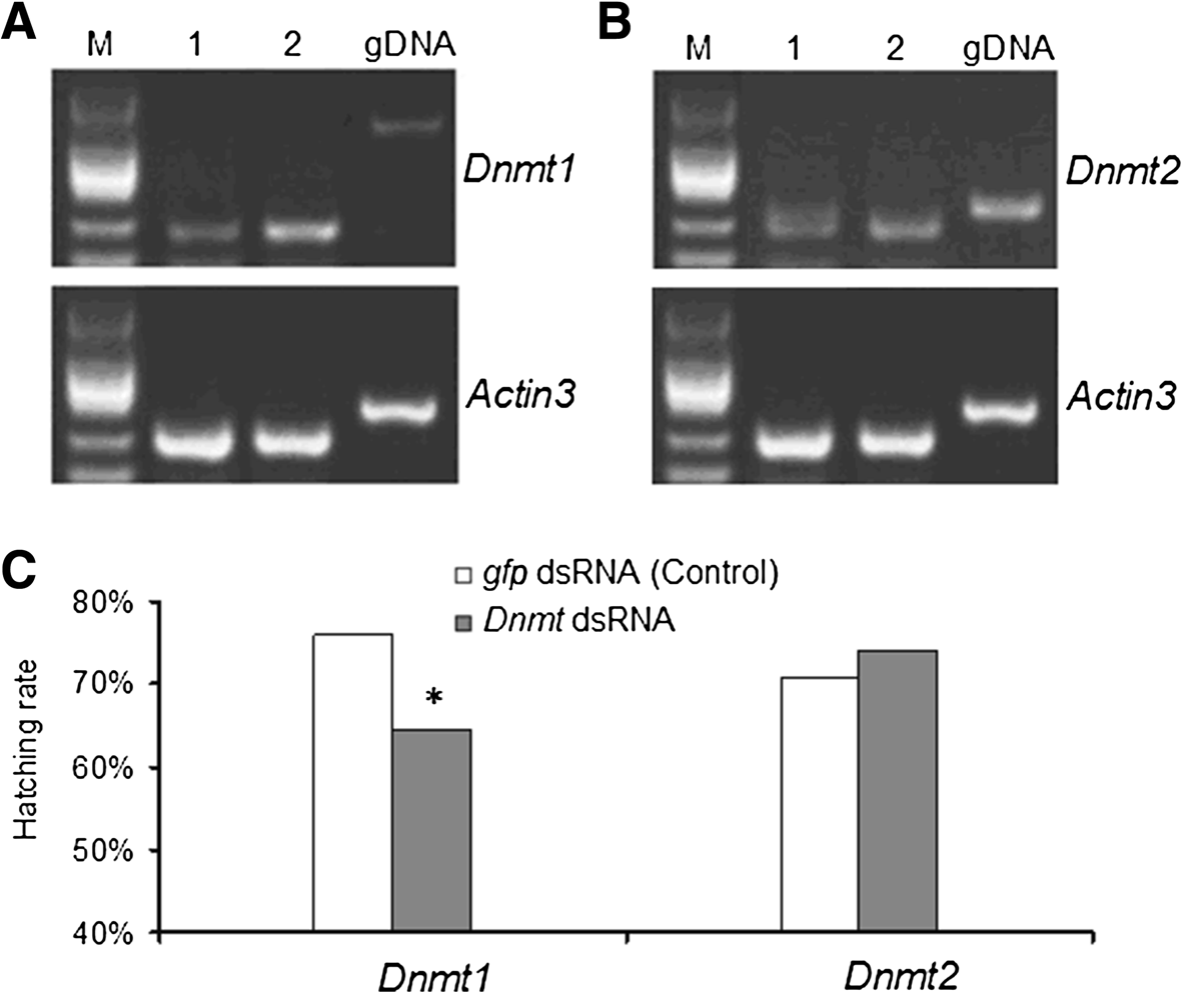
**Summary of RNAi depletion experiments. (A)**, **(B)** Semi-quantitive RT-PCR validation of the effects of RNAi knockdown on the *Dnmt1***(A)** and *Dnmt2***(B)**, indicating obvious decrease of expression level of *Dnmt1***(A)** and *Dnmt2***(B)**. Lane 1 indicate amplification using cDNA from *Dnmt1* RNAi eggs **(A)** and *Dnmt2* RNAi eggs **(B)**, respectively; Lane 2 indicate amplification using cDNA from Non-specific RNAi control (by embryonic microinjection of *gfp* dsRNA) eggs. gDNA, PCR using genomic DNA as template to control DNA contamination; M, DNA marker DL2000 (TakaRa, Japan). *Actin3* is used as the internal control for Semi-quantitive RT-PCR. **(C)** Hatching rate of the treated eggs with *Dnmt1* RNAi and *Dnmt2* RNAi, indicating that RNAi knockdown of *Dnmt1* significantly reduces hatched eggs compared to control, but not for *Dnmt2*. * significant differences as determined by chi-squared test (p < 0.01).

To explore the possible evolution role of DNA methylation, we first test if the two silkworm DNA methyltransferase genes (*Dnmt1*, *Dnmt2*), have biological function. Here using RNA interference (RNAi) we found experimental evidence that *Dnmt1* is important to embryo development. Injections of double strand RNA (dsRNA) of *Dnmt1* and *Dnmt2*, respectively to ~ 8 h eggs led to down regulation of their expression level (Figure 1A). As negative control, injection of *gfp* (green fluorescence protein gene) dsRNA had no obvious effect on the amount *Dnmt1* and *Dnmt2* mRNAs. We found that *Dnmt1* embryonic RNAi resulted in a significant decrease in hatching rate while *Dnmt2* RNAi did not have this effect (Figure 1B). The result of *Dnmt1* suggests a functional importance of this methyltransferase in silkworm embryonic development, congruent with findings on both plants and mammals [18,19]. As to *Dnmt2*, one possibility is that *Dnmt2* doesn’t have functional effect to the embryo development, or our RNAi didn’t work. But it is also possible that because the basal expression of this gene is too low as indicated by Figure 1B, RNAi knockdown may not have obvious effect on the hatching rate of silkworm eggs. Further efforts to completely knockout *Dnmt2* may be able to provide solid evidence on the functionality of *Dnmt2*.

Based on this finding as well as our previous observation that DNA methylation level is positively correlated with gene expression in silkworms [12], our study is extended to the more complex issue of possible epigenetic influences to silkworm domestication. We conducted a comprehensive methylome comparison of silk glands between three domesticated and four wild silkworms. Besides using the previously reported Dazao strain’s methylome [12], we used the MethylC-Seq technology [20] to reveal the silk gland methylomes of other two strains of domesticated silkworms (C108 and JingSong) and four wild, geographically different silkworms. Dazao and C108 are reserved strains and JingSong (Js) is a well-known high silk production strain, while the four wild silkworms were collected from four different geographic areas (Additional file 1: Figure S2): Sichuan (CW), Shaanxi (XW), Yunnan (Wyn) provinces and Shanghai (Wsh), among which Sichuan and Shaanxi are speculated to be the key origins of domesticated silkworms according to current historical records [21]. Information of all individual samples is shown in Additional file 2: Table S1. Our deep sequencing of bisulfite-treated DNAs yielded 2.92 Gb ~ 4.04 Gb effective data for the two domesticated silkworms and 2.84 Gb ~ 3.86 Gb for the four wild silkworms (Additional file 3: Table S2), resulting in 5.31 ~ 5.75 ×, 4.12 ~ 5.61× coverage per strand and 67 ~ 81%, 77 ~ 80% genomic cytosines covered by effective reads in domesticated and wild silkworms, respectively (Additional file 3: Table S2).

Previously, we showed that the silkworm has a sparse methylation genome and cytosines in non-CG contexts are barely methylated [12]. We accordingly referred to our previous procedures [12] to call methylated CGs (mCGs) in each sample and included our published silk gland methylome of the domesticated strain Dazao [12] in the domesticated samples. Out of all CGs with high-quality sequencing support, 224,802 ~ 328,834 and 181,243 ~ 252,685 were found to be methylated in domesticated and wild silkworms, respectively (Additional file 3: Table S2). CG sites covered in both wild and domesticated silkworms (totally 15,920,418) were selected to call for differentially methylated CGs (DMC) and conserved methylated CGs (CMC) between species. mCGs covered in both the species, are 146,003 ~ 201,501 in domesticated silkworms and 119,269 ~ 161,644 in wild silkworms(Additional file 3: Table S2) Based on our stringent criterion (see Methods section) to identify CMCs and DMCs, totally we identified 31,072 CMCs and 6815 DMGs, among which 4,792 DMCs are in domesticated silkworms and 2,023 in wild ones. It is notable that CMCs and DMCs are totally less than 1/3 of the mCGs identified for each samples, and thus large proportion of mCGs do not consistently exist within species, suggesting that, like genetic polymorphism [10], DNA methylation also shows substantial within-species variation. We observed 4,792 DMCs in domesticated silkworms but only 2,023 in wild ones. The 2-fold more DMCs in domesticated silkworms may possibly reflect a trend of increasing DNA methylation during silkworm domestication. Another domesticated species, chickens, also show higher methylation levels than their wild ancestors [22]. Whether this is a general phenomenon during domestication is an interesting issue to investigate in more domesticated organisms.

**Figure 2:**
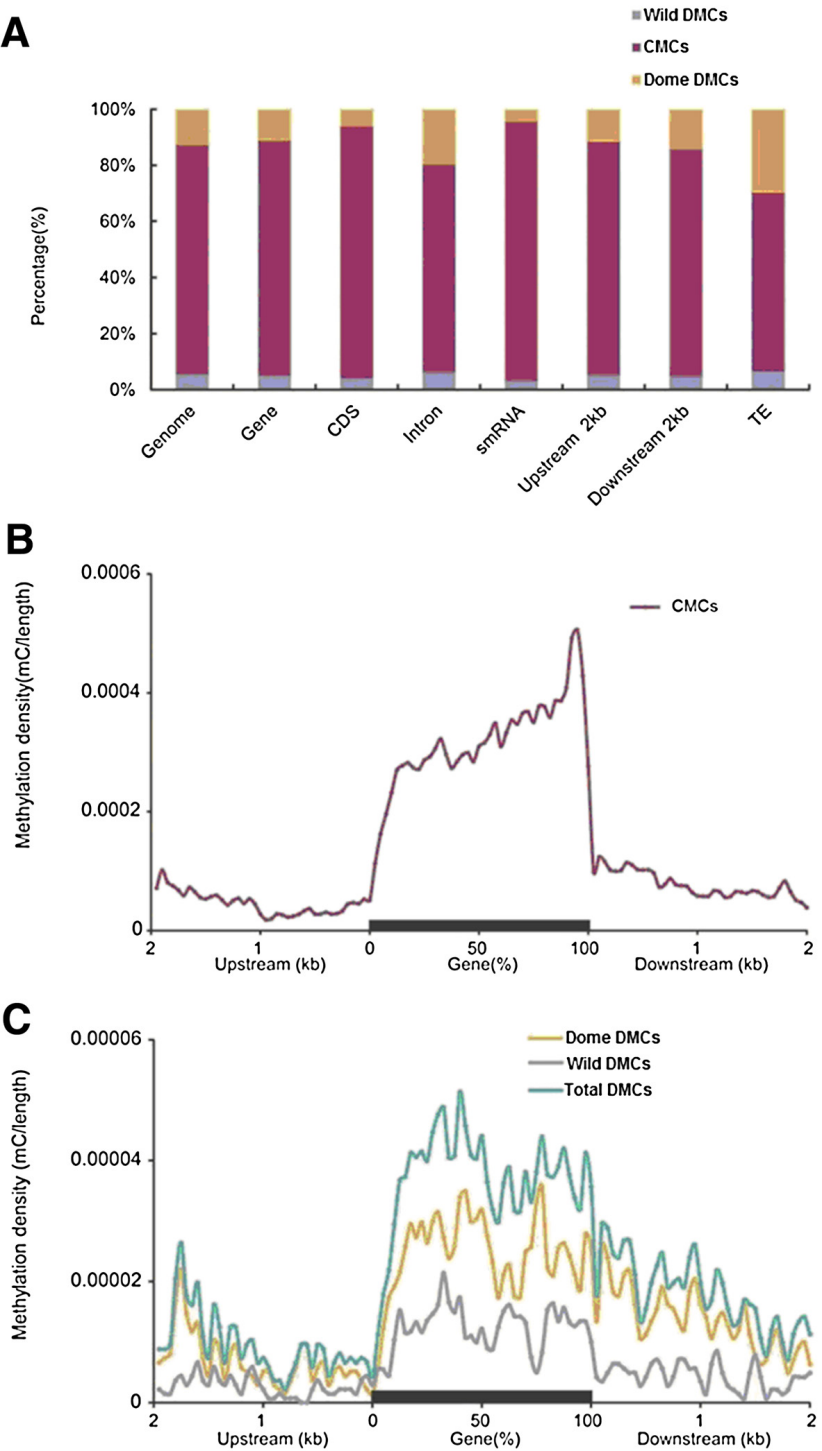
**Distributions of interspecific conserved (CMC) and species differential (DMC) mCs in different functional elements. (A)** Proportion of CMCs and DMCs in different genomic functional regions. **(B)**, **(C)** Analysis of CMCs **(B)** and DMCs **(C)**. Two-kilobase regions upstream and downstream of each gene were divided into 100-base pair (bp) intervals. Each gene was divided into 20 intervals (5% per interval). Plots show methylation density in each interval. Dome, domesticated silkworm specific; Wild, wild silkworm specific.

In contrast to *Arabidopsis thaliana*, in which TE and other repeats show conserved methylation while genic especially coding sequences (CDS) methylation are much more variable either among natural populations or generations [23,24], here we observed that methylation in CDS and smRNA loci in silkworms are apparently conserved between the domesticated and wild silkworms, but TEs and introns bear more methylation differences between the two species (Figure 2A). Consistently, DMCs are significantly enriched in introns (3021 DMCs vs 8731 CMCs) (*p* < 0.01, chi-square test) and in TEs (1066 DMCS vs 1873 CMCs) (*p* < 0.01, chi-square test) compared to in CDS (1963 DMCs vs 17402 CMCs). We used the ant methylome data to test enrichment of DMCs in intron and TEs compared to CDS in two ant species [25] and found similar pattern both in *Camponotus floridanus* (2488 DMCs vs 10133 CMCs in introns (*p* < 0.01, chi-square test) and 261 DMCs vs 1279 CMCs in TEs (*p* < 0.05, chi-square test) compared to 12434 DMCs vs 70698 CMCs in CDS) and in *Harpegnathos saltator* (1945 DMCs vs 13053 CMCs in introns (*p* < 0.01, chi-square test) and in 461 DMCS vs 3431 CMCs in TEs (*p* < 0.05, chi-square test) compared to 7707 DMCs vs 71937 CMCs in CDS). The results suggest DNA methylation in TEs and introns may be more dynamic than CDS in insect species.

We further plotted mCG profiles in the context of gene regions (including introns) and their 2 kb up- and downstream regions. Distribution of CMCs is similar to our previous results [12], higher genic but lower up- and down-stream methylation, with boundaries between gene bodies and flanking sequences showing a sharp drop (Figure 2B). Notably, within each regions CMCs are evenly distributed (Figure 2B) while densities of DMCs show drastic fluctuation across each tested region (Figure 2C), perhaps reflecting the regulatory nature of those DMCs.

Difference pattern in conserved and variable methylated functional regions between plants (*Arabidopsis thaliana* and rice) and insects such as silkworms and ants may indicate that, in contrast to plants where stable DNA methylation for controlling activities of TEs is more important [23,24,26], in insects, maintaining methylation status in genic regions rather than TEs seems predominant [12,27,28], possibly because the functional role of DNA methylation in silkworms is mainly in genic regions. Indeed the functional significance of genic region methylation has been reported and three explanatory mechanisms have been proposed, e.g. keeping accurate transcription [26], regulating alternative splicing [29] or alternative promoter silencing [30], whether these hypothetical explanatory mechanisms are able to address the above phenomena observed in insects requires further experimental evidence.

In total, DMCs are located in 2437 gene bodies, 352 upstream and 670 downstream 2 kb regions of genes. Usually mCGs clustered in a certain region are more likely to have functional implications than single mCG, we further screened for DMC clusters, at a cutoff of 3 DMCs within a 250 bp interval considering sparse mCs in silkworms (0.1% mCs on average) [12]. This kind of DMC clusters was found in 16 upstream, 25 downstream regions and 147 gene bodies (Additional file 4: Table S3). We refer to these regions as gene-related differentially methylated regions (DMR).

**Figure 3:**
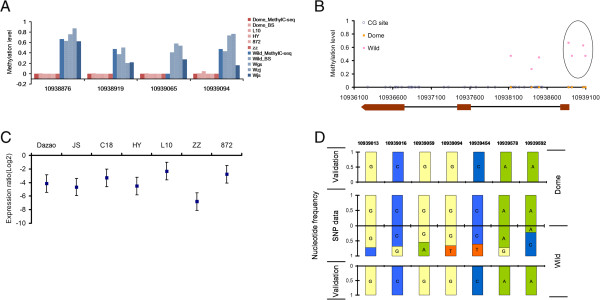
**Methylation and expression of BGIMBGA003527 as well as SNPs in its upstream 2 kb region. (A)** Comparison of MethylC-Seq data of the BGIMBGA003527 DMC cluster with the 454 sequencing data of traditional bisulfite PCR (BS-PCR). Methylation level of the DMCs were examined by MethylC-seq in the three domesticated silkworms Dazao, Js, C108 (Dome_MethylC-Seq) and in the four wild silkworms Wsh, Wyn, CW, XW (Wild_MethylC-Seq) and validated in the same sample sets (Dome_BS and Wild_BS). Validation and test for fixation of methylation differences in more new domesticated (L10_BS, HY_BS, 872_BS, ZZ_BS) and wild silkworms (Wgs_BS, Wzj_BS, Wjs_BS) listed in Additional file 2: Table S1, were conducted using 454 sequencing of BS-PCR amplicons. For MethylC-Seq and validation data, methylation level was calculated by dividing the total reads from each sample set covering mCG by the total reads from that sample set covering that cytosine. For fixation test, methylation level was calculated similarly except that the reads are from each individual. **(B)** Location of DMCs in genic regions. The gene model is at the bottom, where red blocks indicate exons and the black lines between each two blocks indicate introns. The 4 CG sites in the black ellipse are those validated and tested for fixation. Dome, domesticated silkworms, Wild, wild silkworms. **(C)** BGIBMGA003527 expression level changes in silk glands of each domesticated individual against wild silkworms, estimated by quantitative real-time PCR. Relative expression ratios of each domesticated to wild silkworms are normalized by logarithmic transformation. Plots shows the average relative expression ratios and error bars shows the standard errors. **(D)** Nucleotide frequency of the SNPs upstream 2 kb of BGIMBGA003527 surveyed from the published data (http://silkworm.swu.edu.cn/silkdb/resequencing.html) and validated for domesticated and wild silkworms listed in Additional file 2: Table S1 by Sanger sequencing.

**Figure 4:**
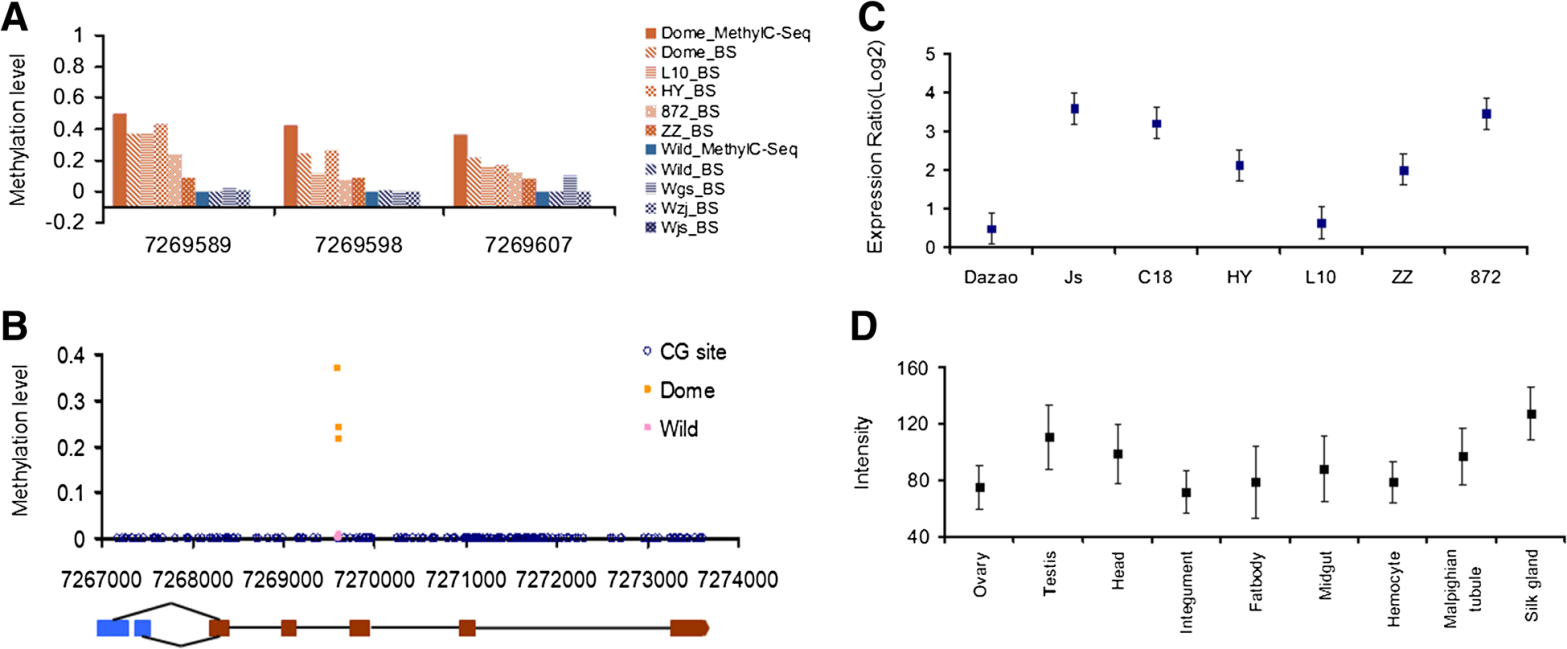
**Methylation and expression of BGIBMGA000155. (A)** Comparison of MethylC-Seq data of the BGIMBGA000155 DMC with the 454 sequencing data of traditional bisulfite PCR (BS-PCR) sequencing. Detail sample and method information is the same as in Figure 3(A). **(B)** Location of the DMC cluster in genic regions. The gene model is at bottom, where blocks indicate exons (red for constitutive exons and blue for alternative exons, respectively) and the black lines between each two blocks indicate introns. Dome, domesticated silkworms, Wild, wild silkworms. **(C)** BGIBMGA000155 expression level changes in silk gland of each domesticated (Dazao, Js, C108, L10, HY, ZZ and 872) individual against wild silkworms (Wsh, Wyn, CW, XW, Wzj, Wjs and Wgs), estimated by quantitative real-time PCR, indicating that this gene is generally up-expressed in the domesticated silkworms. Relative expression ratios of each domesticated to wild silkworms are normalized by logarithmic transformation. Plots show the average relative expression ratios and error bars show the standard errors. **(D)** Expression of BGIBMGA000155 in different tissues of Dazao examined by microarray analysis, indicating this gene has a higher expression level in silk gland. Plots show the average intensities and error bars show the standard errors.

Due to considerable epigenetic instability, gain or loss of DNA methylation is common compared to DNA mutations, which are usually irreversible [23]. The heritable and even fixed epimutations during domestication are thus awash with prompt but reversible epigenetic changes. We therefore decided to further test more domesticated and wild samples in order to find fixed DMR. We collected four more domesticated silkworm strains (three high silk production strains, HY, L10 and 872, and one local reserved strain ZZ) and three more geographically different varieties of wild silkworms from Gansu (Wgs), Zhejiang (Wzj) and Jiangsu (Wjs) province of China (Additional file 2: Table S1, Additional file 1: Figure S2). Unfortunately, checking all the 188 DMRs with loci-specific bisulfite PCR and sequencing (BS-PCR) for these individuals is too costly, so we randomly chose 37 regions to subject to bisulfite PCR amplification followed by 454 deep sequencing, using the barcoded primers (Additional file 5: Table S4). In total, from 25 out of these 37 regions (Figures 3A &4A, Additional file 1: Figures S3 & S4), we successfully obtained effective 454 sequencing data in at least three new domesticated and two new wild samples, among which 12 regions were covered by sequencing data across all the samples. As to the rest 12 regions, although all of them were successfully amplified in domesticated silkworms and 10 out of them had effective sequencing data in at least 3 new domesticated samples, only 5 regions could be amplified in only one new wild sample, leaving us with a lack of informative sequencing data in wild silkworms for these 12 regions. Failure of sufficient amplification in wild samples may be due to mutations in primer binding regions.

Among the 25 DMRs, 5 have consistent DMC clusters between at least two new individuals of both domesticated and wild silkworms, suggesting general fixation of the five DMC clusters. The 5 DMRs include: the upstream regions of BGIBMGA003527 (Figure 3B); introns of BGIBMGA000155 (Figure 4B), BGIBMGA006408 and BGIBMGA002594; and exon of BGIBMGA009144 (Additional file 1: Figure S3). BGIBMGA003527 is of particular interest as all the tested 4 CGs in the DMC cluster at its upstream region show fixed demethylation in domesticated silkworms. It is noteworthy that because DNA methylation is usually cell/cell type specific, we didn’t observed 100% methylation level at the 4 CGs in wild silkworms (Figure 3A). Fine mapping of the DMC cluster indicated that they are just 200 bp upstream of the coding sequences, which could be in 5′UTR or promoter (Figure 3B). Interestingly, BGIBMGA003527 had been identified to be a candidate silkworm domestication gene in a genomic region of selective signal (GROSS) [10]. We conducted more analyses to assess whether the selective sweep in this region is caused by key genetic, or epi-mutations, or even both. To test the genetic mutation, we carefully checked SNPs in BGIBMGA003527 using the available SNP data of 40 domesticated and wild silkworms (http://silkworm.swu.edu.cn/silkdb/resequencing.html) and found that all the four SNPs detected in CDS are synonymous differences and thus do not change the protein sequence (data not shown). The evolutionary process of this gene might thus affect its expression, by affecting on regulatory elements. As expected, BGIBMGA003527 shows lower silk gland expression level in the tested domesticated silkworms than the wild ones (Figure 3C). To test genetic mutations of its upstream regulatory regions, we also used the released SNP data (http://silkworm.swu.edu.cn/silkdb/resequencing.html) and found 7 SNPs in the upstream 2 kb region of BGIBMGA003527 showing obvious different frequencies between domesticated and wild silkworms (Figure 3D). We further sequenced the region covering the 7 SNPs of all the tested domesticated and wild silkworms and interestingly, all the tested domesticated and wild silkworms are of the same genotype in this tested region (Figure 3D). Therefore, the differential expression of the gene between wild and domesticated silkworms is less likely to be caused by genetic mutations in the tested regulatory sequences, indicating that the fixed epigenetic difference is likely to be the cause of the BGIBMGA003527 expression change during silkworm domestication. This finding bears very important evolution significance by indicating that selective sweep may be able to act on epimuations. BGIBMGA003527 is homologous to *Drosophila CG11050*, which was supposed by domain analysis to have the molecular function of metal ion binding and phosphoric diester hydrolase activity. How this function was selected by humans needs more functional studies.

The other four genes were not in the domestication gene list proposed previously [10]. Unlike BGIBMGA003527, the tested DMC clusters within these genes were all proved to have generally higher methylation level in domesticated silkworms (Figure 4A; Additional file 1: Figure S3). Among the four genes, BGIBMGA000155 showed overall silk gland overexpression in domesticated silkworms compared to wild ones (Figure 4C), and microarray analysis of 5th instar larvae of Dazao strain (BmMDB: http://silkworm.swu.edu.cn/microarray/) indicate its higher expression in the silk gland (Figure 4D). BGIBMGA000155 is homologous to the *Drosophila icln* gene. ICLN is a multifunctional protein that is essential for cell volume regulation [31], which invokes a very interesting correlation between its high expression level and enlargement of silk gland in domesticated silkworms.

We did not observe obvious expression changes for the other three genes (Data not shown). BGIBMGA002594 is a silkworm adenylate kinase (ADK) gene. ADK catalyzes the reversible phosphoryl transfer from adenosine triphosphates (ATP) to adenosine monophosphates (AMP) and to yield adenosine diphosphates (ADP). BGIBMGA009144 and BGIBMGA006408 have no functional clue. The former has a WD-40 repeat and the latter has a tetraspanin domain, both of which are found in diverse proteins.

We tested genetic divergence in these genes between the domesticated and wild silkworms by analyzing SNPs identified from the MethylC-Seq data in this study and didn’t detect obvious fixed different SNPs between the tested domesticated and wild silkworms (Additional file 6: Table S5). As for the effect of DNA methylation on gene expression, it seems that epigenetic changes might also be associated with expression changes for the BGIBMGA000155 gene. However, we didn’t observe expression changes for other three genes, which may indicate that their DNA methylation differentiation may not have caused change of gene expression levels.

We only tested 25 regions out of 188 gene-related DMRs identified through comparative methylomics between the 3 domesticated and 4 wild silkworms, and found one DMR has evolved fixed methylation difference while 4 others have largely evolved methylation difference between domesticated and wild silkworms. Extrapolating from these proportions, there may be 7 strictly fixed and 30 generally differentially methylated regions at the whole genome level between domesticated and wild silkworms. Compared to 354 genes strongly selected by humans during silkworm domestication [10], DNA methylation-differentiated loci during domestication are very limited. Understandably, such a reversible epigenetic change may not be able to account for large scale phenotypic evolution, whether under domesticated or natural conditions. It is, however, plausible that some epigenetic changes may provide a trajectory for evolution of new traits [4,8], especially for quick responsive adaptation. Thus artificial selection might act on epigenomes to acquire human-preferred traits [9]. In mammals, reprogramming (demethylation/remethylation) of methylation pattern takes place during two developmental stages in germ cells and in preimplantation embryos [32], whereas in plant germline cells, CG and CHG methylation are usually maintained and only CHG methylation is reprogrammed [33], which may allow transmitting responsive DNA methylation changes to the next generation with higher chance in plants. Even in mammals, transgenerational inheritance of epialleles has also been reported [34-37]. In insects, before fertilization, one oocyte is divided into one germline cell and seven nurse cells, which are similar to plants but different from a mammalian oocyte. Accordingly, although insects have multitude lower methylation, if their germline reprogramming process is like that in plants, epialleles are still more likely to be transmitted to the next generation.

## Conclusions

Though much future research is needed to provide additional experimental evidence that can confirm our observation regarding epigenetic divergences and their possible contribution during silkworm domestication, our study has provided highly suggestive experimental evidence on the functional importance of the epigenetic system in silkworm and candidate genes that might be associated with epigenetic changes during domestication. Further functional exploration of these genes may lend vital evidence regarding the role of epigenetic contribution in artificial selection. In addition, this study has also given impetus to the case for *Dnmt1*’s role in DNA methylation in insects.

## Methods

### Silkworms and sample preparation

Two domesticated silkworm (*B. mori*) strains and four geographically different individuals of wild silkworm (*B. mandarina*) were used for MethylC-Seq [12,20]. We also included the domesticated strain Dazao MethylC-Seq data (Biological duplicate 1 because of the higher genome coverage and depth covered by MethylC-seq data, (http://ftp.genomics.org.cn/silkworm_methylation) for the comparative analysis. Furthermore, we included another four domesticated strains and three wild individuals to narrow down interspecific differential methylation by bisulfite-PCR and differential expression by RT-PCR. Detailed information of these individuals is shown in Additional file 2: Table S1. The silk glands of 5th-instar larva of each individual of the silkworms (*B. mori* and *B. mandarina*) were ground into powder in liquid nitrogen, respectively. Half of the powder from each silk gland was used to extract total DNA using DNeasy Blood and Tissue Kit (Qiagen), and the other half was used to extract total RNA using RNeasy Mini Kit (Qiagen).

The research protocols on all animal experiments has been reviewed and approved by the internal review board of Kunming Institute of Zoology, Chinese Academy of Sciences. (approval ID: SYDWLLWYH-20100909-001).

### Procedure for embryonic RNAi knockdown of the silkworm *Dnmt1* and *Dnmt2*

Silkworm non-diapause strain *Nistari* was used as the material. Larvae were reared at room temperature and fed on mulberry leaves.

RNA was extracted following the above method. Total RNA was digested with DNase I (Takara) to remove remaining DNA. Complementary DNA (cDNA) was synthesized using the RevertAid First Strand cDNA Synthesis Kits (Fermentas). Primers designed to amplify suitable regions were: 5′-CTCACTCTGCGAGCTTTGT-3′ (forward) & 5′-GTCGTCGTAGCGATACTGTAG-3′ (reverse) for *Dnmt1*; 5′-ATGATTACTTGGTGCCAGAC-3′ (forward) & 5′-ATACTCTTATTCATCAAACAA-3′ (reverse) for *Dnmt2*. dsRNA of *gfp* was used as non-specific control and primers designed for a 439 bp DNA fragment of the *gfp* gene were TGGTGAGCAAGGGCGAGGAG (forward) & TCGTCCATGCCGAGAGTGAT (reverse). The plasmid pEGFP-N1pPIGA3GFP (Invitrogen, USA) was used as template to amplify *gfp* gene. For the production of a template to synthesize double strand RNA (dsRNA), fragment of the each of the three genes were amplified using above specific primers respectively, with a T7 promoter sequence (5′-TAATACGACTCACTATAGGG-3′) at the 5′ end of each primer. dsRNA was prepared using the MEGAscript RNAi kit (Ambion, USA) according to the manufacturer’s protocols. Sense and antisense transcripts were simultaneously synthesized using 1 μg template in one reaction. dsRNA concentration was about 600 ng/μl.

Silkworm eggs were kept 7 h after oviposition and were then used for microinjection of dsRNA with the microinjection system (Leica, Germany; Narishige, Japan). The injection amount was approximately 10-20 nl. Injection of equivalent volume of *gfp* dsRNA was used as control. Totally, more than 400 eggs were injected with dsRNA of *Dnmt1* and *Dnmt2* and 220 eggs with *gfp* dsRNA. The whole microinjection process lasted for about 1 h. The injected eggs were incubated at 25°C in a moist Petri dish placed in a sealed container where humidity was maintained by a water immersed lens cleaning tissue.

Nearly 24 h after injection, about 20 eggs of each treatment were sampled to extract RNA and synthesize cDNA using the above method. Semi-quantitative RT-PCR were applied to test the effect of RNAi knockdown of *Dnmt1* and *Dnmt2* with primers Dnmt1f (5′-CTCTCTCCGATGGCACTAAGT-3′) & Dnmt1r (5′-ATAGCCGACCGTAGAGCC-3′) for the former and Dnmt2f (5′-TTTACAGCGGTATTGGTGG-3′) & Dnmt2r (5′-TGGCAAGGTGGTGACATGAG-3′) for the latter. Expression of the silkworm *Actin3* gene was set as an internal control. *Actin3* was amplified using the primers A3f (5′-GCTCGAACAGTGCGCATT-3′) & A3r (5′-GATACCTCTTTTGCTCTGTGCC-3′). Hatched eggs were recorded approximately 10 days after injection, and lasted 3 days thereafter, given that a full proportion of eggs in control treatments had hatched.

#### Methylome sequencing

MethylC-Seq library construction and sequencing; mapping and initial processing of MethylC-Seq reads; mC identification and removal of background noises were described in our previous study [12].

#### Identification of differentially methylated cytosines (DMCs), conserved methylated cytosines (CMCs)

For each CG site that both domesticated and wild silkworms’ reads covered, if methylated CGs were in at least three individuals of one species but not more than one individual in the other species, then the CG site was recorded as a DMC. Methylated CGs in at least three individuals of both domesticated and wild silkworms was recorded as a CMC.

#### Real-time PCR

Total RNA was digested with DNase I (TaKaRa) to remove the remaining DNA. Complementary DNA (cDNA) was synthesized using the RevertAid First Strand cDNA Synthesis Kits (Fermentas). Expression of BGIBMGA003527and BGIBMGA000155 were validated by Real-time PCR using primers 5′-AAGACTTGGACCGTTATGAT-3′ (forward) & 5′-GACAACGGTATGTTTCTCAA-3′ (reverse) for the former and 5′-ATGGGGCGGAGGCGTTAGT-3′ (forward) & 5′-ATTCGTTCTCGTTTTCTGGGAT-3′ (reverse) for the latter. Real-time PCR was performed in two duplicates with SYBR Green PCR Mix (Bio-Rad) and subjected to the Roche LightCycler 480 Real-Time PCR System.

#### Bisulfite-PCR validation and fixation test for selected DMCs using 454 sequencing

One microgram of genomic DNA from the silk gland of each individual listed in Additional file 2: Table S1 was bisulfite-converted according to our previous method [12]. Primers were designed to amplify target regions covering the selected DMC clusters and modified according to the requirement for 454 amplicons sequencing. Primers were also barcoded by adding a specific index sequence in their 5′ regions. For validation of MethylC-seq data, the equal aliquot of amplicons from domesticated silkworm sample set (Dazao, Js, C108) and the wild silkworm sample set (Wsh, Wyn, CW, XW) were barcoded respectively while for fixation test, amplicons were individually barcoded. Equal aliquot amplicons were pooled to construct 454 sequencing library according to the manufacturer’s instruction (454 Life Sciences, Branford, CT, USA). Eventually we obtained 25.8 Mb sequencing data. BLAST searches (e-value <1e^10-3^) against the original target sequence database were performed to map the raw reads. Matched sequences with length ≥100 bp were used for further calculation of methylation level at each single cytosine site. All the primers were listed in Additional file 5: Table S4.

#### SNP survey and validation by Sanger sequencing

The SNP data of 40 silkworms (29 domesticated and 11 wild silkworms, respectively) (http://silkworm.swu.edu.cn/silkdb/resequencing.html) and gene annotation data (http://silkworm.swu.edu.cn/silkdb/doc/download.html) were obtained from the SilkDB. SNPs located in CDS and upstream 2 kb of BGIBMGA003527 were retrieved and calculated for nucleotide frequency. The 7 SNPs detected in the upstream 2 kb of BGIBMGA003527 were further validated in the silkworms listed in Additional file 2: Table S1. The region covering the SNPs were amplified using the primers 5′-AATCTTTGTAAATGCCTGAC-3′ (forward) & 5′-TTATTCTGTCCAATTTAGTAGG-3′ (reverse). PCR products were sequenced using an ABI 3700 DNA Analyzer (ABI PRISM).

#### Microarray analysis

The normalized microarray data of the BGIBMGA000155 were obtained from the *B. mori* microarray database (BmMDB: http://silkworm.swu.edu.cn/microarray/download.html) and calculated for average intensity in each tissue.

#### Identification of CMCs and DMCs between queens and workers in two ant species, Camponotus floridanus and Harpegnathos saltator

Methylome data of the two ant species, namely *Camponotus floridanus* and *Harpegnathos saltator* were obtained from NCBI GEO database (GSE31576). For each species, we further analyzed CMCs and DMCs between queens and workers, according to Bonasio et al.’s methods [25] with some modifications.

#### SNP identification from MethylC-Seq data

SNP calling from MethylC-Seq reads was conducted using the Bis-SNP package (http://epigenome.usc.edu/publicationdata/bissnp2011/). The SNP database of 40 silkworms (29 domesticated and 11 wild silkworms, respectively) (http://silkworm.swu.edu.cn/silkdb/resequencing.html) used in this package were obtained from the SilkDB. For each SNP locus, if at least one domesticated and one wild silkworm has nucleotide information, then this locus retained as effective one for further analyses.

### Accession codes

Methylome data have been deposited into the NCBI Short Read Archive (SRA, http://www.ncbi.nlm.nih.gov/sra/) under the accession number SRA062224.

## Abbreviations

RNAi: RNA interference; dsRNA: Double strand RNA; mCGs: Methylated CGs; DMC: Differential methylated CGs; CMC: Conserved methylated CGs; CDS: Coding sequences; DMR: Differentially methylated regions; BS-PCR: Bisulfite PCR and sequencing; GROSS: Genomic region of selective signal.

## Competing interests

The authors declare that they have no competing interest.

## Authors’ contributions

WW, HX and JW designed the study. HX, WW, and AW wrote the manuscript. XL and GJZ developed the method for mapping and processing BS reads. XX constructed the MethylC-seq libraries and did the high throughput sequencing. FYD and QYX provided the silkworm samples and detail background information on silkworm domestication and breeding. QYL and JML analyzed the ant methylome data. AJT and QHG conducted the microinjection. LC and YD did the BS-PCR validation and SNP test. XL and HX analyzed the MethylC-Seq Data and 454 data. XL identified SNPs of the silkworms by MethylC-Seq data. HX and QHG extracted DNAs and RNAs and did RT-PCR and Real-time PCR. All authors have read and contributed to the manuscript.

## Supplementary Material

Additional file 1: Figures S1-S4 **and the cited references **[15,28,38-41]**.** Click here for file

Additional file 2: Table S1 Details of domesticated and wild silkworm samples. Click here for file

Additional file 3: Table S2 Summary of sequencing results and reads alignment for three domesticated and 4 wild silkworms. Click here for file

Additional file 4: Table S3 Summary of DMC clusters. Click here for file

Additional file 5: Table S4 PCR primers used to amplify methylation differentiated genes. Click here for file

Additional file 6: Table S5 SNP sumary of the five condiate DMRs identified by MethylC-Seq reads. Click here for file
