# Supplementary material for: Comparative methylomics between domesticated and wild silkworms implies possible epigenetic influences on silkworm domestication
Source: BMC Genomics. 2013 Sep 23;14:646. doi: 10.1186/1471-2164-14-646 (PMC3852238; doi:10.1186/1471-2164-14-646)
Supplement: Additional file 1: Figures S1-S4 — and the cited references [15,28,38-41]. [file 1471-2164-14-646-S1.doc]

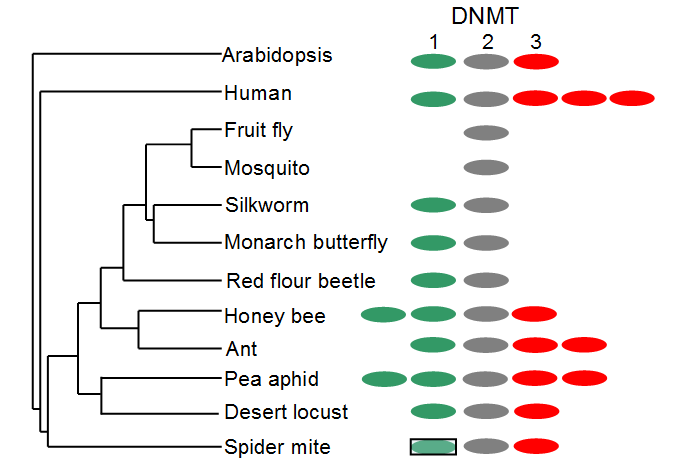


**Figure S1. Distribution of DNA methyltransferase genes in human, Arabidopsis, arthropods including red spider mite and insect genomes.** The figure is modified from Schaefer et al [38] and Feng et al [15]. The number of ellipses of each color represents the number of gene copies found in each species. Updated information includes: monarch butterfly [39]; ant [40]; pea aphid [28], desert locust; red spider mite [41]. In this species, *Dnmt1* are suspected to have become to be a pseudogene, as indicated with a blank rectangular.

**
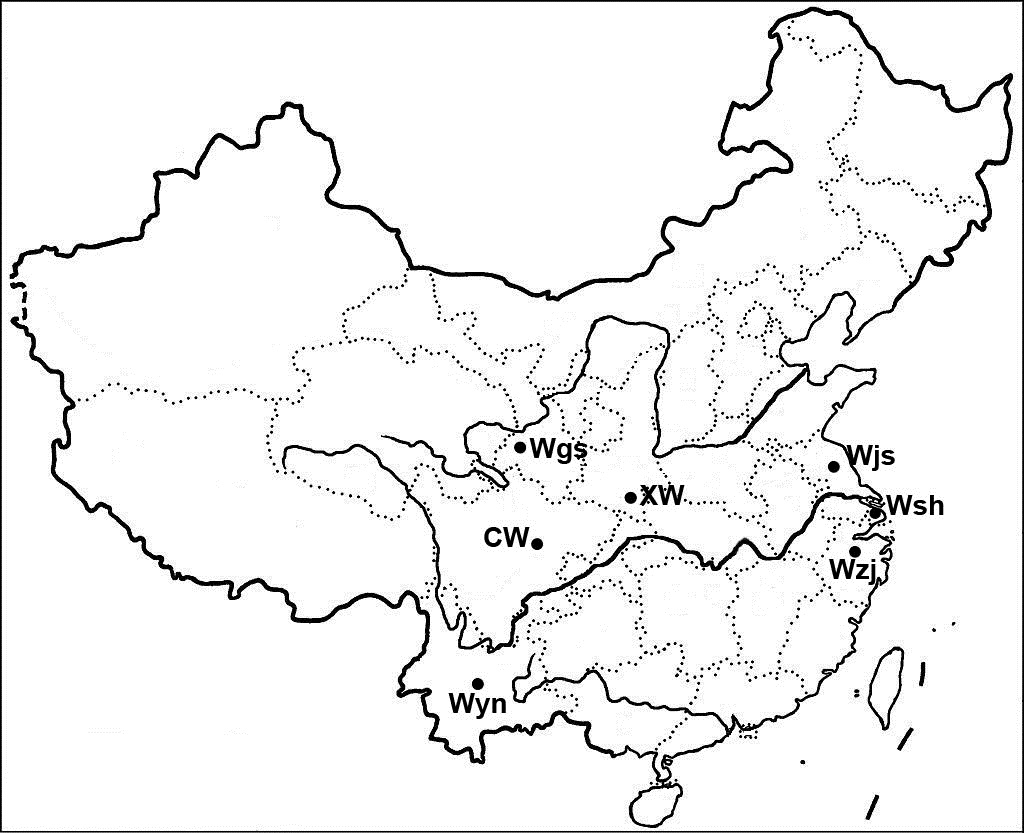
**

**Figure** **S2.** **Geographic locations of the wild silkworm individuals used in this study.**

**Figure** **S3. Methylation of three representative genes.** Comparison of MethylC-Seq data of the DMC cluster in BGIMBGA002594 **(a)**, BGIMBGA009144 **(b)** and BGIMBGA006408 **(c)**, with the 454 sequencing data of traditional bisulfite PCR (BS-PCR). Methylation level of the DMCs were examined by MethylC-seq in the three domesticated silkworms Dazao, Js, C108 (Dome_MethylC-Seq) and in the four wild silkworms Wsh, Wyn, CW, XW (Wild_MethylC-Seq) and validated in the same sample sets (Dome_BS and Wild_BS). Validation and test for fixation of methylation differences in more new domesticated (L10_BS, HY_BS, 872_BS, ZZ_BS) and wild silkworms (Wgs_BS, Wzj_BS, Wjs_BS) listed in table S1, were conducted using 454 sequencing of BS-PCR amplicons. For MethylC-Seq and validation data, methylation level was calculated by dividing the total reads from each sample set covering mCG by the total reads from that sample set covering that cytosine. For fixation test, methylation level was calculated similarly except that the reads are from each individual. Location of DMCs in genic regions is on the right of each panel. The gene model is at bottom, where blocks indicate exons (red for constitutive exons and blue for alternative exons, respectively) and the black lines between each two blocks indicate introns. Dome, domesticated silkworms, Wild, wild silkworms.

**
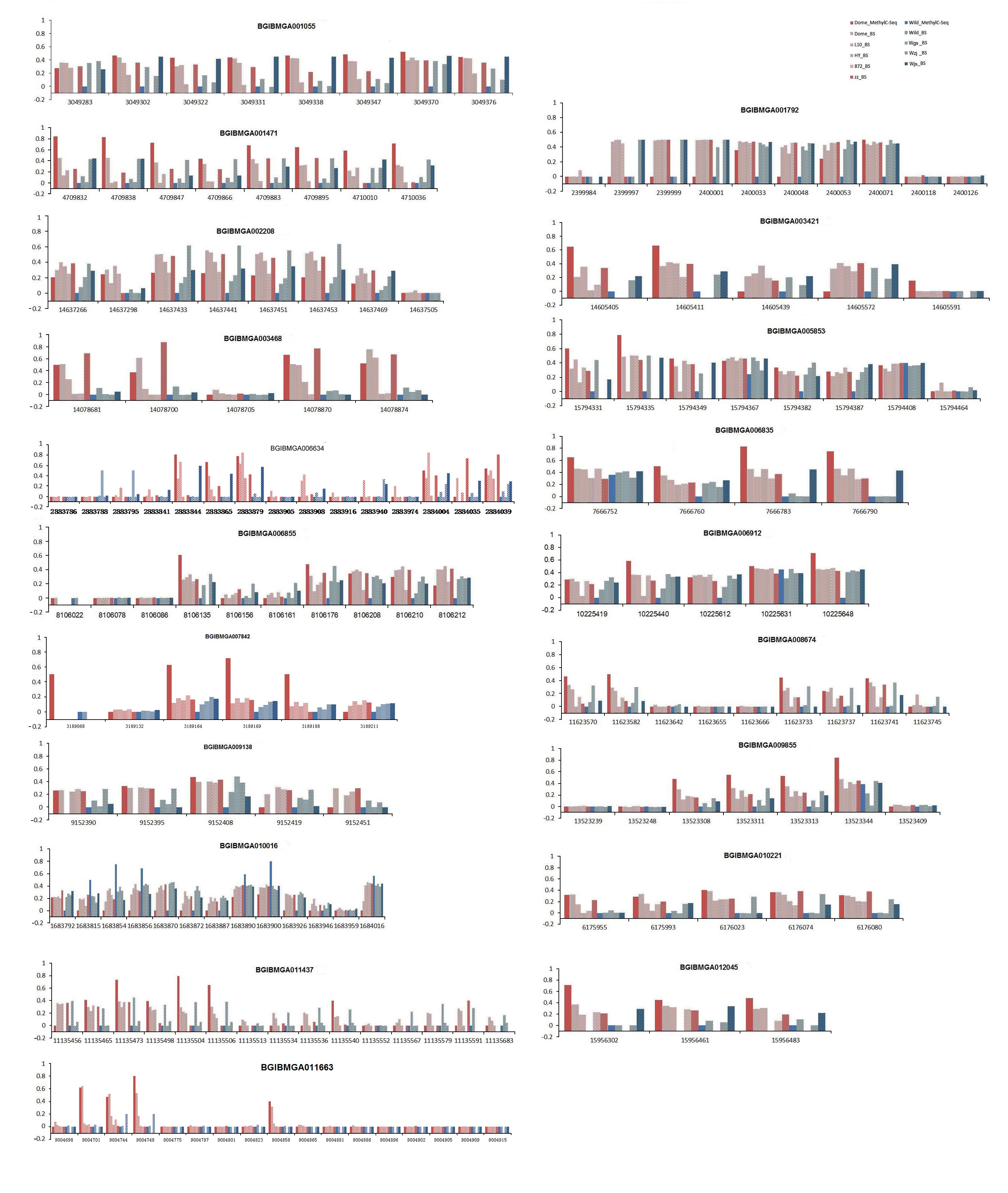
**

**Figure** **S4. Comparison of MethylC-Seq data of the DMC cluster in the other 20 related genic regions with the corresponding 454 sequencing data of traditional bisulfite PCR (BS-PCR).** Detail information was described in Supplementary Fig. S3.

15. Feng S, Jacobsen SE, Reik W: **Epigenetic reprogramming in plant and animal development**. *Science* 2010, **330**(6004):622-627.

28. Walsh TK, Brisson JA, Robertson HM, Gordon K, Jaubert-Possamai S, Tagu D, Edwards OR: **A functional DNA methylation system in the pea aphid, *Acyrthosiphon pisum***. *Insect Mol Biol* 2010, **19 Suppl 2**:215-228.

38. Schaefer M, Lyko F: **DNA methylation with a sting: an active DNA methylation system in the honeybee**. *Bioessays* 2007, **29**(3):208-211.

39. Zhan S, Merlin C, Boore JL, Reppert SM: **The monarch butterfly genome yields insights into long-distance migration**. *Cell* 2011, **147**(5):1171-1185.

40. Bonasio R, Zhang G, Ye C, Mutti NS, Fang X, Qin N, Donahue G, Yang P, Li Q, Li C *et al*: **Genomic comparison of the ants *Camponotus floridanus* and *Harpegnathos saltator***. *Science* 2010, **329**(5995):1068-1071

41. Grbic M, Van Leeuwen T, Clark RM, Rombauts S, Rouze P, Grbic V, Osborne EJ, Dermauw W, Ngoc PC, Ortego F *et al*: **The genome of Tetranychus urticae reveals herbivorous pest adaptations**. *Nature* 2011, **479**(7374): 487-492.
